# Circulating Tumor Cells for the Monitoring of Lung Cancer Therapies

**DOI:** 10.3390/ijms27010384

**Published:** 2025-12-30

**Authors:** Maja Chrzempiec, Urszula Oleksiewicz

**Affiliations:** 1 Department of Cancer Immunology, Poznan University of Medical Sciences, Rokietnicka Street 8, 60-806 Poznan, Poland; 2 Department of Diagnostics and Cancer Immunology, Greater Poland Cancer Center, Garbary Street 15, 61-866 Poznan, Poland

**Keywords:** circulating tumor cells (CTCs), lung cancer, liquid biopsy, prognostic marker, predictive marker

## Abstract

Lung cancer, as one of the most prevalent and lethal malignancies, requires immediate and effective therapeutic solutions. Therefore, additional innovative methods are continually sought to achieve optimal treatment outcomes. Various markers are used to select the most effective therapies, assess clinical responses, and facilitate follow-up care for the patients. Circulating tumor cells (CTCs) remain a valuable biomarker in clinical management of cancer patients due to the range of information they provide and their high prognostic and predictive potential in monitoring anticancer therapy. CTCs constitute a heterogeneous population of cancer cells that undergo an epithelial-to-mesenchymal transition (EMT), are shed from the tumor mass, and migrate through the peripheral blood, ultimately causing metastases. In this literature review, we focus on the biological, biochemical, and biophysical properties of CTCs, specifically from the perspective of the design of CTC enumeration technologies. Furthermore, we combine the available data on the application of CTC count in monitoring various treatment modalities in lung cancer, including radiotherapy, chemotherapy, tyrosine kinase inhibitors, and immunotherapy. Although many published reports indicate that an increased number of CTCs in blood samples of lung cancer patients correlates with worse treatment outcomes, several limitations hinder the widespread usage of CTCs in the clinical setting.

## 1. Introduction

Lung cancer is the most frequent cancer in both sexes globally, with the highest incidence and mortality rate. According to the Globocan 2022 report, approximately 2.48 million new cases and 1.82 million deaths were reported worldwide, while the 5-year prevalence affected 3.22 million people. It accounted for 12.4% of all new cancer cases and 18.7% of cancer-related deaths [1]. Based on the Global Burden of Disease (GBD) 2019 study, trachea, bronchus, and lung cancer (TBL) accounts for 18.1% of all cancer-related Disability-Adjusted Life Years (DALYs). The vast majority of the DALYs burden for TBL cancer was due to Years of Life Lost (YLLs), which accounted for 98.8%, while Years Lived with Disability (YLDs) accounted for only 1.2% [2]. The frequent causes of lung cancer include: radon, asbestos, long-term and cumulative exposure to air pollution, and personal or familial lung cancer history. However, the most common cause is cigarette smoking, which accounts for up to 80% of fatal cases [3]. In general, in 2021, tobacco accounted for 29.32% of all cancer-related DALYs in males and 8.09% in females [4].

Based on the microscopic appearance, lung cancer is classified into non-small cell lung cancer (NSCLC) and small cell lung cancer (SCLC). NSCLC, diagnosed in 85% of all lung cancer patients, is further classified into squamous cell carcinoma, adenocarcinoma, and large cell lung cancer. SCLC, in turn, occurs in the remaining 15% lung cancer cases. This type of cancer is particularly dangerous because it grows quickly and causes metastases, which is why it is often diagnosed in the late stages associated with a worse prognosis [3].

Numerous treatment options may be offered to a lung cancer patient, depending on tumor status and patient condition. Apart from surgical resection, the treatment strategy may include radiotherapy, chemotherapy, kinase inhibitor therapy, immunotherapy, or a combination of selected therapies. To ensure its efficacy, it is crucial to adequately monitor the response to treatment. Monitoring approaches can be divided into physical and molecular techniques. Physical methods include imaging modalities (e.g., computed tomography or magnetic resonance imaging) and pathological examination (e.g., bronchoscopy or sputum cytology) [5]. Particular attention is paid to the molecular profiling of cancer samples, including those collected via liquid biopsy. A liquid biopsy allows for the retrieval of body fluids (blood, saliva, or bronchial lavage fluid) using minimally invasive approaches. The components of a liquid biopsy, such as free nucleic acids or circulating tumor cells (CTCs), may provide vital information to guide the clinical management of the patient. CTCs are cancer cells that detach from primary and metastatic tumors, enter the bloodstream, and migrate through peripheral blood into distant locations, potentially initiating the formation of secondary tumors. Due to these properties, CTCs can be utilized as a reliable marker of disease progression and an indicator of treatment effectiveness [6]. Growing evidence indicates that high CTC levels before and after the initiation of treatment are associated with poor prognosis in lung cancer treatment [7,8,9,10,11]. Therefore, many studies concentrate on the development of efficient CTC enrichment techniques, their molecular profiling, and assessment of their utility in the clinical setting [6].

This literature review aims to present the outcomes of clinical experiments that employ CTC enrichment methods to predict responses to lung cancer therapies. The following sections provide an overview of CTC biology, as well as the benefits and limitations of various enumeration techniques. Moreover, we will revise available data on the application of CTCs in clinical trials investigating the impact of different lung cancer treatment modalities, including radiotherapy, chemotherapy, tyrosine kinase inhibitors, and immunotherapy.

## 2. Circulating Tumor Cells

### 2.1. CTCs Biology

Circulating tumor cells (CTCs) were first discovered in 1869 by Thomas Ashworth [12] and further characterized as tumor cells that shed from existing malignant lesions (primary and metastatic) [13,14]. The tumor mass may contain cells of different clonal origin with distinct genetic, epigenetic, and metabolic signatures. Such heterogeneity facilitates survival upon exposure to various stressors (e.g., hypoxia, therapies) and, subsequently, the expansion of optimally adapted clones [15]. It also supports phenotypic plasticity, including the epithelial-to-mesenchymal transition (EMT) and the reverse process, mesenchymal-to-epithelial transition (MET). Both phenomena are crucial mechanisms occurring during metastasis and are closely linked to CTC biology. During EMT, a cancer cell loses its epithelial features and acquires a mesenchymal state. The cells exhibit decreased E-cadherin expression and increased expression of Vimentin and N-cadherin. Mesenchymal cells exhibit higher migratory and invasive properties, which facilitate their shedding from the tumor mass and penetration into the lymphatic and blood vessels. As CTCs circulate with the blood flow, they may extravasate the blood vessel and spread into distant sites in the body, thus causing metastasis [16]. CTCs most often spread directly through blood vessels. However, CTCs may also enter lymph vessels and disseminate through lymphatic system into lymph nodes [17,18]. In patients with melanomas, carcinomas, and sarcomas, the first route of metastasis is usually through the tumor-draining lymph nodes (TDLNs). In patients with lung, breast, and colon cancer, approximately 29–37% of patients are diagnosed with lymph node metastasis [19]. Colonization of the metastatic niche occurs through the MET pathway. The cells undergoing MET lose their migratory potential and change their phenotype to epithelial. They may remain dormant for a period of time or initiate proliferation leading to the outgrowth within the metastatic site [16]. These attributes highlight the CTC potential for clinical application in monitoring tumor progression [20].

As a consequence of phenotypic plasticity, the CTC population may be heterogeneous, with cells exhibiting epithelial, intermediate/hybrid, and mesenchymal phenotypes. The epithelial type is characterized by apical-basal polarization and high expression of epithelial markers, e.g., EpCAM (Epithelial Cell Adhesion Molecule) and cytokeratins (CKs). These traits are typical for primary tumor cells, which retain strong intercellular connections. The mesenchymal type, in turn, is characterized by reduced expression of epithelial markers and increased expression of mesenchymal markers. The cells in the mesenchymal state exhibit a greater ability to migrate and invade other tissues [21]. Multiple factors may drive invasion. For example, metalloproteinases secreted by CTCs and other tumor-interacting cells degrade the extracellular matrix (ECM), thereby facilitating detachment from the primary tumor, traversal through the ECM, intravasation, and the subsequent reverse process: extravasation [22]. The intermediate phenotype reflects the incomplete transitions between epithelial and mesenchymal (E/M) states [21,23]. It exhibits markers characteristic of both states [15,16]. Notably, E/M hybrids represent an array of metastable intermediate states characterized by high plasticity, allowing for dynamic transitions towards more epithelial or mesenchymal states [23]. These cells exhibit increased viability in the detached state, a higher potential for collective migration and metastasis, as well as more apparent stem cell-like characteristics [21,23,24,25]. Indeed, the data indicate that CTCs may express both EMT and stem cell markers [26,27]. Notably, CTCs with stem-like features represent a subpopulation with increased survival, chemoresistance, aggressiveness, and tumor-initiating capacity within the metastatic niche [13,27].

EMT may be induced by a number of transcription factors [e.g., TWIST family of basic helix–loop–helix factors (TWIST), Snail Family Transcriptional Repressors (Snail1/2), Zinc-finger E-box-binding homeobox factors (ZEB1/2)] and signaling pathways [e.g., Transforming Growth Factor-β (TGF-β), Wnt, Notch], acting via distinct yet frequently interconnected molecular mechanisms. For example, Snail1 and Snail2 are zinc-finger transcription factors that bind to regulatory E-box motifs within the *E-cadherin* promoter, ultimately leading to the transcriptional repression of *E-cadherin*. ZEB1 and ZEB2 also bind to the same E-box but recruit histone deacetylase Sirtuin-1, causing histone deacetylation and repression of *E-cadherin* [21,23,28,29,30]. Furthermore, the Warburg effect, a hallmark phenomenon in cancer cells, may also evoke EMT [21]. Warburg effect refers to a metabolic remodeling, in which cancer cells preferentially rely on aerobic glycolysis over oxidative phosphorylation (OXPHOS) for energy production. This phenomenon results in increased lactate levels and reduced production of reactive oxygen species (ROS). Such situation appears when tumor cells are in specific conditions, e.g., hypoxia or low pH. Various studies have shown that under these conditions, the phenotype of cancer cells may change due to EMT induction. For example, high glucose levels, loss of fructose-1,6-bisphosphatase, or epidermal growth factor (EGF) may augment glycolysis, thus contributing to EMT. Moreover, the Warburg effect has a protective role over CTCs. In metastasis progression, there is a high risk of excessive production of ROS, which can lead to anoikis—cellular death associated with detachment. By limiting the flux of the mitochondrial OXPHOS pathway, the Warburg effect reduces ROS production in mitochondria, thereby protecting cancer cells from anoikis during dissemination. Furthermore, large amounts of lactic acid secreted by tumor cells inhibit the activation of Natural Killer (NK) cells and T lymphocytes, which results in decreased immunological surveillance [21].

### 2.2. CTCs Interactions in the Bloodstream

For the metastatic cascade to occur, cancer cells must switch their phenotype and overcome several barriers related to the intravasation into the vasculature, circulation, and subsequent dissemination and colonization at distant sites. The first barrier is the tightness and adhesion of the cells and extracellular matrix surrounding the tumor mass. When the tumor cells overcome the adhesion forces, they may enter the bloodstream. Often, along with this process, the surrounding tissues and matrix become degraded. Cells within the vasculature are constantly subjected to various obstacles: shear stress due to high blood pressure [31], the risk of anoikis evoked by loss of attachment, and attack from immune cells. Fluid shear forces may trigger mechanical damage to the majority of CTCs, leading to their subsequent elimination [32]. However, shear stress was also demonstrated to mediate the survival of a small subpopulation of CTCs that exhibit an increased EMT phenotype, stemness, and metastatic potential [32,33,34,35]. Another threat is immune system cells, which catch and eliminate harmful cells that could cause metastases. However, CTCs develop numerous defense mechanisms, thereby effectively escaping immune surveillance [30]. A key feature contributing to the successful retention of CTCs in the bloodstream is their ability to maintain a cluster structure [30]. A small subset of CTCs enters the vasculature as multicellular clusters composed of tumor cells [36]. Moreover, they may form heterotypic clusters through interactions with other cells within their circulome, including platelets, neutrophils, macrophages, myeloid-derived suppressor cells (MDSCs), or cancer-associated fibroblasts (CAFs) [13,37].

Circulome is a collection of various cellular, subcellular, and molecular entities circulating in the bloodstream [38]. In addition to blood cells, the circulome of oncology patients may also contain components derived from cancerous tissue: CTCs, circulating tumor DNA (ctDNA), and tumor-derived extracellular vesicles. Platelets, which directly interact with CTCs, play a significant role in circulome. They may safeguard CTCs against various stress factors, such as shear forces and immunological surveillance (e.g., exposure to NK cells). The clotting cascade is initiated by coagulation-associated genes expressed by CTCs (e.g., Tissue factor), which triggers thrombin generation. Platelets can form aggregates with CTCs in two scenarios. The first one refers to the complete engulfment of circulating cancer cells. In the second one, platelets form homotypic aggregates in the center of the clusters, which are surrounded by cancer cells on the periphery. In both scenarios, platelets protect CTCs against adverse conditions by forming so-called “platelet-rich thrombi” [38,39].

Furthermore, platelets were shown to contribute to EMT by the synergistic activation of TGFβ/Smad and Nuclear Factor Kappa B (NFκB) pathways triggered by direct contact between platelets and cancer cells. Labelle and colleagues [40] conducted a study on mice, implanting them with colon carcinoma cells (MC38GFP) and breast carcinoma cells (Ep5), which had been previously coincubated with purified platelets to investigate the potential for metastasis. The results showed a significant increase in the number of tumor cells detected in the lungs at 48 h post-injection and subsequently a higher number of metastatic foci after 14 days, compared to the cells that were not treated with platelets. Notably, prior exposure to platelets was associated with an increased mesenchymal phenotype and invasiveness in in vitro experiments [40].

In addition, the platelet-cancer cell clusters promote the involvement of other immune cells, such as macrophages, by releasing various chemokines, for example, C-X-C Motif Chemokine Ligand (CXCL12). Interactions between cancer cells and immune system cells occur in the bloodstream and in the tumor microenvironment (TME). Although CTCs may be targeted by immune cells (e.g., NK cells), they also cooperate with macrophages, which can lead to immune evasion. In TME, cancer cells may promote the recruitment of inflammatory monocytes, which differentiate into macrophages. Macrophages and other immune cells present in TME secrete various chemokines, cytokines, and growth factors that increase the permeability of blood vessels, facilitating the migration of tumor cells through blood vessels. Furthermore, macrophages promote invasion and metastasis by engaging tumor cells in an autocrine loop via EGF signaling. Tumor cells are stimulated to produce macrophage colony-stimulating factor (M-CSF), which, in turn, induces EGF release by macrophages, and the whole cycle continues [41].

Macrophages, together with neutrophil extracellular traps (NETs), also facilitate the extravasation of CTCs from blood vessels into the extracellular matrix to establish metastases [41]. Adhesion of CTCs and neutrophils is possible via interaction of integrin Mac-1 on the surface of neutrophils and Intercellular Adhesion Molecule 1 (ICAM-1) on the surface of CTCs [42]. Such interaction facilitates the CTC anchorage to the blood vessel endothelium and extravasation into the surrounding tissue via the release of Interleukin-1β and matrix metalloproteinases [13,43]. In addition, neutrophils, by their physiology, can form NETs—web-like structures consisting of DNA and proteins released from activated neutrophils. The published data indicate that NET formation facilitates the capture of CTCs in the bloodstream, invasion through endothelial monolayers in vitro, and metastatic dissemination [44]. Neutrophils were found as the main cell type within CTC-white blood cell clusters in breast cancer patients. This interaction promoted the expression of genes involved in cell cycle progression, supporting the survival and metastatic potential of CTCs compared to solitary CTCs [37]. Furthermore, neutrophils may interfere with the activation of anti-tumor response from various immune cells, such as NK cells [43], peripheral leukocytes [45], and cytotoxic T cells [46]. This inhibitory effect facilitates immune evasion by CTCs.

Another group of cells that influence CTCs are myeloid-derived suppressor cells (MDSCs), which constitute a heterogeneous population of immature myeloid cells produced in the bone marrow. They mainly inhibit the anti-tumor activities of T lymphocytes and NK cells, hence promoting both tumor growth and metastasis. Polymorphonuclear-MDSCs (PMN-MDSCs), a subpopulation of MDSC, may form clusters with CTCs. Sprouse and colleagues [47] conducted a study on mice by co-injecting luciferase-labeled breast cancer MDA-MB-231BR (Brc-luc) cells or melanoma Lin-negative/CTC-enriched cell populations into the systemic circulation, along with patient-matched PMN-MDSCs. Their results showed a significantly higher number of metastases and spread to the lungs and brain when co-injecting tumor cells with PMN-MDSCs as compared to animals injected with tumor cells or PMN-MDSC alone. Moreover, heterotypic clusters composed of PMN-MDSCs and CTCs were detectable in the peripheral blood and bone marrow of mice for an extended period. These observations suggest that PMN-MDSCs not only exhibit immunosuppressive abilities but also promote metastasis and increase CTC survival in the bloodstream [47]. Indeed, a high level of PMN-MDSCs within the vasculature of tumor patients, but not within the tumor mass, is associated with shorter recurrence-free survival after surgery, as shown in NSCLC [48].

In addition to other cell types described above, cancer-associated fibroblasts (CAFs), as one of the most dominant cellular components of the TME, also have the ability to interact with CTCs. They are involved in various cancer-related processes, including tumorigenesis, metastasis, immunosuppression, drug resistance, and metabolic reprogramming. By releasing numerous factors, such asTGF-β, CXCL12, and Interleukin 6, CAFs can promote EMT. CAFs can also drive cancer cell invasion by forming channels in the extracellular matrix (ECM). Moreover, similarly to MDSCs, CAFs can form protective clusters at the center and front of CTCs [49,50,51].

In conclusion, CTCs constitute a significant risk for disease dissemination. However, their detrimental impact is facilitated mainly by the support of other circulome components, including platelets, immune cells, and CAFs, which collectively enhance EMT, survival in the bloodstream, and extravasation into distant tissues.

### 2.3. Biological, Biochemical and Biophysical Characteristics of CTCs

**Figure 1:**
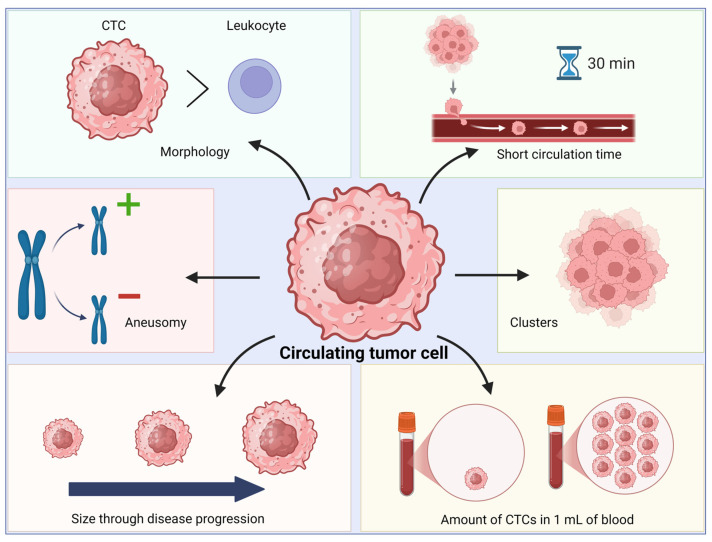
Basic CTC features. CTCs exhibit characteristic morphology with a granular or stippled nucleus. Moreover, they have a higher nuclear-to-cytoplasmic ratio and are larger in size compared to white blood cells. Frequently, CTCs demonstrate aneusomy (addition [+] or deletion [-] of chromosomes) and an increased size of single CTCs during disease progression. They have low abundance, as 1–10 CTCs may be detected in 1 mL of blood in patients with metastatic disease. CTCs may form clusters with intercellular connections in the bloodstream, which can consist of up to 100 CTCs. They also have a short circulation time—25–30 min for a single CTC. Created in Biorender.com (http://BioRender.com/9ep189q), accessed on 15 December 2025.

In addition to their transient phenotypic states, CTCs exhibit a range of biological features that could be leveraged in the design of effective CTC detection methods (Figure 1). Generally, CTCs exhibit an increased size compared to white blood cells [52]. However, this is not always the case. The diameter of the smaller CTC subpopulation (around 9 µm) falls within the range of leukocyte diameter (7–9 µm) [53,54]. The data indicate that CTC sizes vary across different types of tumors. For example, the median CTC diameter in the case of breast cancer is higher (12.4 µm) than in bladder cancer (8.6 µm) and colorectal cancer (7.5 µm) [55]. Moreover, due to their phenotypic heterogeneity, CTCs within a blood sample may reveal different sizes. A study on advanced lung cancer [51] identified two CTC subpopulations based on their size: large cell CTCs, with diameters of 20–30 µm, and small cell CTCs, with diameters of 2–4 µm. Many small CTCs expressed vimentin, indicating a mesenchymal phenotype, and their presence correlated with a poor prognosis [56].

Another characteristic parameter is a high nuclear-to-cytoplasmic ratio. According to the observation at 90° angles, the ratio of nuclear size (diameter) to cytoplasmic size (diameter) in CTCs has a mean value of 0.8 compared to 0.55 in the case of white blood cells. Moreover, the CTC nucleus is usually granular or stippled. Meng and colleagues [57] compared the sizes (diameters) of CTCs in different stages of breast cancer. The measurement of 50 CTCs per patient revealed that the mean cell size of CTCs was 29.8 µm in patients with dormant disease, 33.9 µm in patients with metastasis, and 32 µm in patients with primary tumors. Furthermore, fluorescence in situ hybridization (FISH) analysis using chromosome enumerator probes allowed for the examination of chromosomes (1, 8, and 17) in every cell at the early stage of the study, and later stages included chromosomes 3 and 11. The observations revealed aneusomy in CTCs. Based on mathematical modeling, the researcher also estimated that the half-life of CTCs in patients with primary tumors could last a few hours [57].

As mentioned in the section above, CTCs show high phenotypic plasticity and heterogeneity. Besides their transitioning between epithelial and mesenchymal states, CTCs may also exhibit variability in survival potential. The study performed by Rossi and colleagues [58] demonstrated that the CTC population consists of both apoptotic and viable cells, and their ratio may dynamically shift during therapy. The researchers used a monoclonal antibody specific for the M30 neoepitope, which appears upon caspase cleavage of cytokeratin 18 (CK18) during the early stages of apoptosis. M30-positive, early apoptotic cells were detected in over 70% of CTC-positive cancer patients before treatment. Moreover, the ratio between M30-positive and -negative CTCs was shown to dynamically change during therapy, which could be potentially utilized as an indicator of active disease [58].

Furthermore, CTCs are capable of forming homotypic multicellular clusters, defined as clusters, comprising more than two tumor cells. In some cases, CTC clusters may contain over 100 tumor cells and remain in the bloodstream with preserved intercellular connections. They provide mechanical cell cohesion, multicellular anteroposterior polarization, cytoskeletal synchronization among interacting cells, and juxtacrine signaling through cadherins and gap junctions. They can also provide mechanical coupling (mechanocoupling) via desmosomes. The retention of intercellular adhesion facilitates coordinated behavior, such as collective directional movement [39,59,60]. Key features of CTC clusters include rarity and high metastatic potential [36,39,59]. The research conducted by Aceto and colleagues [36] showed that despite the low abundance of CTC clusters in the bloodstream, they are more resistant to apoptosis and their metastatic potential is approximately 23–50 times higher compared to solitary CTCs. Moreover, the study revealed that CTC clusters, stabilized by the junctional protein plakoglobin, are likely oligoclonal groups of tumor cells, rather than intravascular aggregates [36,39,59,61]. Although the available data indicate that the majority of CTC clusters arise from the collective migration of tumor cells, CTC aggregate formation may also occur [61]. The data published by Taftaf and colleagues [62] indicated that intercellular ICAM1-ICAM1 interactions facilitate CTC aggregation and adhesion to endothelial cells, which further promotes trans-endothelial migration as evidenced in the in vitro and in vivo experiments [62]. As mentioned previously, apart from homotypic clusters, CTCs may form heterotypic clusters with other cells present in the vasculature (platelets, CAFs, immune system cells), which stimulate the aggressive phenotype of CTCs [63].

Finally, CTCs are rare components of the bloodstream. The estimated number of CTCs in the circulation of patients with advanced cancer is one CTC per one billion blood cells. Furthermore, because of short circulation time (25–30 min for a solitary CTC and 6–10 min for cell clusters) and high apoptotic potential, the majority of CTCs exhibit relatively low metastatic efficiency. In a 1 mL blood sample, it is possible to detect 1–10 CTCs in patients with metastatic disease, and in non-metastatic stages, the amount is reduced by half [36,64]. Therefore, CTC isolation and counting may be difficult due to the low amount in the bloodstream and the short lifespan.

## 3. CTC Enrichment and Analysis Methods

Over the years, various methods and markers have been developed to monitor the effects of treatment and the progression of cancer. A liquid biopsy enables the isolation of several molecules from body fluids, such as blood, plasma, serum, sputum, urine, saliva, or bronchial lavage fluid. Due to its lower invasiveness, liquid biopsy may be successfully applied [6] as an alternative to tissue biopsy. Various biological entities may be evaluated in liquid biopsy samples, including CTCs, circulating tumor DNA (ctDNA), microRNA, cell-free DNA (cfDNA), tumor-educated platelets, or exosomes [65]. CTCs offer significant advantages over other liquid biopsy components due to their downstream applicability for cell culture, genomic and transcriptomic profiling, single-cell analysis, FISH, chromosomal tests, and other analyses [66,67].

**Figure 2:**
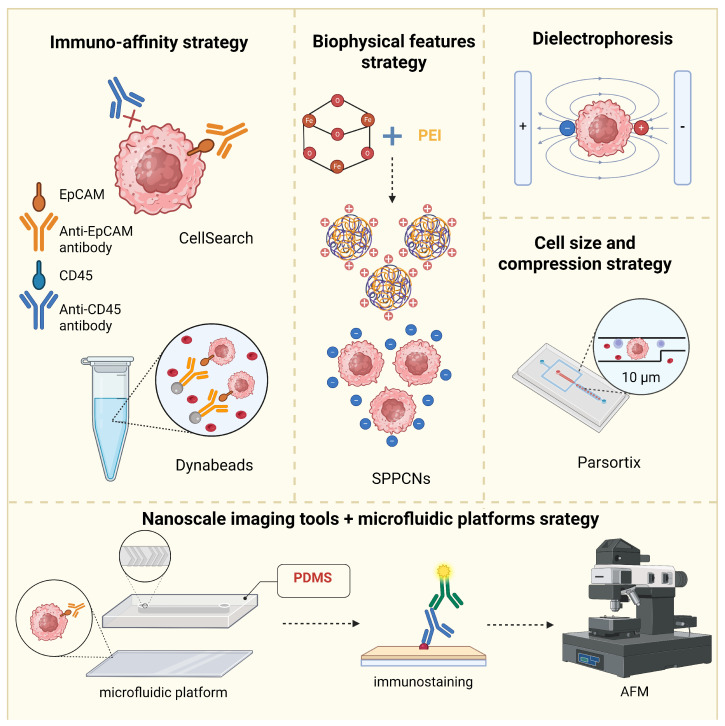
CTC enumeration methods. Illustration of CTC enrichment methods based on immune-affinity strategy (CellSearch, Dynabeads), negative CTC surface charge (SPPCNs), dielectrophoresis, cell size and compression strategy (Parsortix) and nanoscale imagining tools combined with microfluidic platforms strategy. Anti-EpCAM antibody (orange) and EpCAM antigen binding represents key feature in immune-affinity strategy (CellSearch and Dynabeads). PEI denotes a positively charged poly(ethyleneimine) coating of Fe_3_O_4_ nanocore for the capture of negatively charged CTCs. Opposite poles in dielectrophoresis shows polarization between cell membrane and medium that induces dipole moment. In Parsortix last, 10 μm wide channel enables for isolation based on cell size. Bottom chip of microfluidic platform functionalized with antibodies against EpCAM (orange antibody) and top chip composed with polydimethylsiloxane (PDMS) implement immunostaining (with primary [blue] and secondary [green] antibody) and atomic force microscopy (AFM) analysis. Created in Biorender.com (http://BioRender.com/kg7kx54), accessed on 15 December 2025.

Liquid biopsy is gaining popularity due to its ease of collection, non-invasiveness, and reproducibility. Moreover, compared to traditional biopsy, it may facilitate the real-time monitoring of the tumor. Nevertheless, the use of liquid biopsy may be compromised by low marker content, a short cellular half-life, a lack of standardization, and high costs. Due to these limitations, several strategies have been developed to improve CTC enrichment [65]. These methods are based on biological markers (e.g., CellSearch, Dynabeads) utilizing specific antibodies, physical properties (e.g., Superparamagnetic Positively Charged Nanoparticles: SPPCNs, Dielectrophoresis, Parsortix), or a combination of both aspects (Table 1, Figure 2).

### 3.1. Immunoaffinity Strategy

As mentioned above, CTCs are a heterogeneous population of cells; hence, they may present different morphological features, molecular phenotypes, sizes, shapes, densities, and biomarker signatures. The most commonly used biomarker for detecting CTCs is Epithelial Cell Adhesion Molecule (EpCAM or CD326), which is widely expressed in epithelial tumors and does not occur on the surface of hematopoietic cells [74,75]. EpCAM, as a functional antagonist to E-cadherin, disrupts E-cadherin-dependent adhesion by untying β-catenin-F-actin junction, leading to the loosening of cell–cell adhesion and promoting cell movement, proliferation, and metastasis [76]. Early immunoaffinity methods were based on Dynabeads—magnetic beads coated with antibodies against specific cell surface antigens [77]. In 1993, Hardingham and colleagues [78] isolated tumor cells from blood using Dynabeads for EpCAM-based immunomagnetic separation, coupled with a PCR assay for the detection of *K-RAS* codon 12 mutation [77,78].

The positive selection of the EpCAM marker is also utilized in the CellSearch system, the first and only Food and Drug Administration (FDA)-approved assay for CTC enrichment [79]. The test was developed by Veridex LLC (Raritan, NJ, USA) in 2004 to quantitatively measure the level of CTCs from a 7.5 mL blood sample. The entire system consists of patented elements: the CellPrep system (a semi-automated sample preparation system), the CellSearch Epithelial Cell Kit (featuring epithelial cell-specific EpCAM antibodies), and the CellSpotter Analyzer (a four-color semi-automated fluorescence microscope) [68]. Another marker, used for a negative selection, is CD45, a leukocyte-specific protein that is not expressed on CTCs. Altogether, the criteria for CTC identification in the CellSearch assay include round to oval morphology, visible nucleus stained with DAPI, positive EpCAM and cytokeratin (8+, 18+, and/or 19+) staining, and negative CD45 staining [43].

Allard et al. [68] found that the detection frequency of CTCs (greater than two cells) using CellSearch may differ across patient cohorts with various types of malignant tumors. For example, in the lung cancer population, CTCs were detected in 20% of samples (34 of 168 blood samples from patients with metastatic lesions) [68]. The advantages of CellSearch include standardization, reproducibility, analytical performance, flexibility in preparation, and a wide range of clinical applications. Nevertheless, the CellSearch system also has some limitations. Cells are isolated in low quantities, making detection challenging and requiring more sensitive methods, particularly in patients without metastasis. Furthermore, due to CTC heterogeneity, morphological evaluation may be subjective [79]. Additionally, not all CTCs express EpCAM. For instance, EMT leads to the reduced EpCAM expression. A study conducted by Gorges and colleagues [80] showed that in blood samples from mice with metastatic tumors, CTCs were mostly negative for EpCAM but positive for mesenchymal markers (e.g., Twist, Vimentin). In such cases, detection with the CellSearch kit may be inaccurate. Indeed, combined CTC immunomagnetic enrichment, which captures both epithelial EpCAM and mesenchymal N-cadherin, increased the total CTC count by three-fold in a study on advanced ovarian cancer compared to sole EpCAM-based isolation [80,81].

Another problem that may be related to immune-based techniques is the specificity of the antibody used for capturing CTCs. In their blood spike-in experiments, Antolovic and colleagues [69] employed Dynabeads coated with two different monoclonal antibodies specific for EpCAM (BerEP4 and KS1), as well as the HT29 human colon cancer cell line, which expresses EpCAM and cytokeratin 20 (CK20). After immunomagnetic enrichment, RNA was extracted from the immobilized HT29 cells to verify the CTC detection efficiency using an RT-PCR assay for CK20 expression. A study found that in whole blood samples (5 mL), Dynabeads coated with the KS1/4 antibody were able to detect 10^3 HT29 cells (200 cells/mL), whereas the BerEP4 antibody had a ten times lower detection efficiency. Moreover, prior mononuclear cell fractionation with Ficoll gradient centrifugation enhanced the sensitivity of CTC detection [69]. A wide range of immunoaffinity-related technologies has been developed over the years, including AdnaTest, Gilupi CellCollector, MagSweeper, CTC-ChIP, and others [69].

### 3.2. Strategy Utilizing Cell Surface Charge

Apart from antigen-based approaches, several other solutions were devised to improve the CTC enumeration process and minimize its limitations. These technologies utilize various biological and biophysical properties of CTCs. Certain CTC enumeration strategies were developed based on the Warburg effect. CTCs are characterized by a significantly increased rate of glycolysis [82]. The level of glucose uptake and lactate secretion in cancer cells can be up to 30 times higher than in normal cells. Thus, the continuous secretion of lactate anions removes positive ions from the cell surface, leaving a negative charge on the cell membrane. This feature differentiates CTCs from other cells, which typically have an electrically neutral or slightly positive surface charge [82].

In 2020, Wu et al. [70] developed a separation method based on superparamagnetic positively charged nanoparticles (SPPCNs). These nanoparticles consist of ${Fe}_{3}O_{4}$ nanocore covered with an amorphous silica shell. Initially, these nanoparticles have a strong negative charge. However, functionalization with poly(ethyleneimine) (PEI) molecules provides a strong positive surface charge. Due to electrostatic interactions, CTCs bind to SPPCNs, which can be further separated with a magnet. Then, immunofluorescence staining or immunostaining with fluorescence in situ hybridization (iFISH) is used for the identification of characteristic parameters, such as EpCAM+, CD45-, and the presence of a nucleus (DAPI+). The study revealed that the SPPCNs method may detect 2–8 CTCs per 1 mL of blood from all 25 colorectal cancer patients analyzed, whereas only 0–1 CTCs were detected in healthy donors. This method overcomes the limitations of the EpCAM-based detection method because it does not rely on the expression of epithelial proteins, but on the negative surface charge of cancer cells [70].

### 3.3. Strategy Based on Dielectric Properties

Another method for CTC isolation is dielectrophoresis (DEP) [83]. DEP is a label-free technique that differentiates cells based on their physical features, including size, nuclear morphology, cell membrane morphology, as well as the dielectric properties of cells and their membrane surface. In DEP, cells are suspended in medium with defined dielectric properties and then subjected to an electric field. This procedure induces a dipole moment along the direction of the field. There are two variants of the DEP method. Negative dielectrophoresis (nDEP) occurs when the electrical polarizability of cells is lower than that of the surrounding medium. The DEP force acts in the opposite direction to the electric field gradient. Thus, cells move toward a region of a weaker electric field. In contrast, positive dielectrophoresis (pDEP) occurs when the electrical polarizability of the cell exceeds that of the medium. The DEP force acts in the same direction as the electric field gradient, drawing cells towards regions of stronger electric field [83].

One of the first studies describing CTC enrichment using DEP [84] demonstrated that the dielectric properties of metastatic human breast cancer cells (MDA231) were significantly different from those of erythrocytes and T lymphocytes. This trait enabled efficient separation of tumor cells from the mixture with blood cells. In this protocol, mixed cells were injected into an electric field chamber and washed using an eluate of 10 mS/m conductivity. Cancer cells were selectively trapped under the influence of a DEP force and then released from the chamber, yielding a separation purity of 95%. This method is non-invasive and independent of antibodies, surface proteins, or other markers, thus offering a major advantage over antigen-related technologies. However, its limitation lies in the requirement for precise control of physical conditions [84]. DEPArray and ApoStream are well-established DEP-based technologies available on the market [85].

### 3.4. Strategies Based on Cell Size and Compression

Certain CTC detection technologies are based on cell size and compressibility. ISET, CellSieve, ClearCell, CTC-iChIP, and Parsortix are exemplary CTC isolation platforms that exploit these traits for CTC enrichment [85]. CTC-iChip system as a CTC-specific antigen independent method, depletes cells that are normally present in blood, mainly leukocytes. The system consists of two chips. CTC-iChip1 separates nucleated cells (CTC, leukocytes) from erythrocytes and platelets based on size by array of posts with a pillar size. Then, in CTC-iChip2, leukocytes are labeled with the antibodies against CD45 and CD66b (i.e., the markers specific for leukocytes) and attached to magnetic microbeads. Then, the leukocytes are negatively depleted via magnetophoresis. The method demonstrated high efficiency achieving 97% recovery rate of rare cell population with a sample processing speed of 8 mL of whole blood per hour (up tp 10^7^ cells per second) [86]. Over time, the CTC-iChip system has been improved to simplify operation, automate processes, and reduce production costs. It has a monolithic construction integrating multiple previously separate microfluidic components onto a single chip: deterministic lateral displacement (DLD), inertial focusing stage 1 (IF1), magnetically activated cell sorting stage 1 (MACS1), inertial focusing stage 2 (IF2), and magnetically activated cell sorting stage 2 (MACS2). The procedure involves mixing a blood sample with a buffer containing magnetic beads conjugated with antibodies targeting CD45, CD16, and CD66b, which are specific to white blood cells (WBC). The pre-labeled sample is further injected into the chip. The nucleated (CTCs, WBCs) are separated from non-nucleated cells (red blood cells, platelets) within the DLD area. Then, the nucleated cells are aligned in the IF1 module and magnetically sorted in the MACS1 stage for partial WBC depletion. Finally, the remaining cells undergo another round of purification in the IF2/MACS2 stages. Such a system achieves high efficiency (median 99.5% recovery of added cells within a range of 19 to 5000 cells per mL of blood) and throughput (15–20 million cells per second) [87]. Parsortix, in turn, employs microfluidic technology with a disposable cassette, where cells flow through a series of channels, leading to a final 10 μm wide terminal gap. Most blood cells pass through this aperture, while larger cells, such as CTCs, are retained within the cassette. The main advantages of this system are epitope independence (enabling the capture of CTCs without EpCAM expression), simple operation, low cost, and easy cell release from the cassette. Moreover, it does not require magnetic beads that may cause errors in subsequent analyses. However, a major disadvantage of Parsortix is distinguishing CTCs from leukocytes, which may have similar sizes. Therefore, further identification with specific molecular markers (e.g., cytokeratin, CD45, and DAPI staining) is necessary [88].

Over the years, the method has been improved. Saini et al. conducted a comparative study for isolating CTCs from NSCLC patients using the Parsortix system. The NCI-H1975 cell line was labeled with fluorescent dyes: CellTracker Green CMFDA and the nuclear dye Hoechst 33342, for easier identification. Next, the cells were mixed with blood samples from healthy patients and isolated using the Parsortix system. After that, the researchers performed “in-cassette staining” (PR1) using specific fluorescently conjugated antibodies against cytokeratins (PanCK, CK19), EpCAM, Vimentin (Vim), and CD45, as well as the nuclear stain Hoechst. The study demonstrated that the Parsortix PR1 in-cassette staining method successfully captured single CTCs with epithelial (PanCK+, CK19+, EpCAM+, Vim-, CD45-) or both epithelial and mesenchymal markers (PanCK+, CK19+, EpCAM+, Vim+, CD45-) from blood samples. The system was able to isolate both single cells and cell clusters [89].

### 3.5. Nanoscale Imaging Tools Combined with Microfluidic Platforms

In recent years, there has been an increasing interest in microfluidic techniques. Deliorman et al. developed an atomic force microscopy (AFM)-compatible microfluidic platform for CTCs enrichment and further characterization. The platform consisted of two non-permanently connected, key elements. The top chip was composed of polydimethylsiloxane (PDMS), which formed channels arranged in a herringbone pattern. The lower glass substrate was functionalized with antibodies against EpCAM, Prostate-Specific Antigen (PSA) and Prostate-Specific Membrane Antigen (PSMA), enabling selective binding CTCs from prostate cancer patients. After blood sample insertion, CTCs were captured due to antigen–antibody binding. Then, channels were washed, and immunostained (DAPI+, CK+, EpCAM+) to differentiate them from white blood cells. The AFM tip was further applied to assess the nanomechanical properties of the captured cells. [73]. AFM as a leading nano-imaging technique enables to investigate structure, adhesion, stiffness, and biological functions of various biomolecules, as well as whole cells. It monitors distance-dependent interaction forces and reconstructs tridimensional morphology of the given sample [90]. As it was used in the above study, after peeling-off the top chip, authors measured AFM tip-cell interactions (force-displacement curves). This allowed to determine the elasticity, deformation and cell adhesion forces. The data on prostate CTCs revealed reduced stiffness of metastatic cells compared to the CTCs from localized cancer. These observations confirm previous studies indicating that CTCs with high metastatic potential exhibit increased deformability [73]. However, there are other techniques based on AFM. AFM-force spectroscopy (AFM-FS) allows for simultaneous, quantitative mapping of morphology and nanomechanical properties of cells. Nanoindentation combined with microfluidics is useful for sorting and determining forces driving interactions between CTCs and ECM. Infrared nanospectroscopy (AFM-IR) enables multimodal nanoimagining of morphology, mechanical and chemical properties with a resolution of up to approximately 1 nm, which is helpful in identifying specific biomarkers to differentiate types of cancer cells. Furthermore, High-Speed AFM (HS-AFM) allows for video-rate image acquisition to visualize dynamic processes related to carcinogenesis [90]. The expanding AFM-based technological portfolio show high promise for the nano-scale characterization of the cells. Nevertheless, it remains to be further developed in terms of sample standardization or integration with imaging techniques, to be feasibly used as a reliable CTC investigation tool.

The choice of enrichment method is based on several factors and research assumptions. Label-dependent methods are based on markers (e.g., EpCAM). Due to the possible loss of EpCAM marker in EMT, these methods are characterized by high specificity but low sensitivity. However, label-independent methods based on physical properties in general have high sensitivity and low specificity. Thus, it is important to consider benefits and tradeoffs of the method of choice. An alternative approach may be to combine both frameworks or use the latest single-cell technologies, which allow determining heterogeneity of the tumor [91]. For example, single cell-genomic analysis uses Next-Generation Sequencing (NGS) of single cells, which allows obtaining complete CTC genome and comparing it with primary and metastatic tumors. Other procedures, such as single-cell transcriptomics, qPCR, epigenomic or proteomic analyses may facilitate markers identification and therapy personalization. All the above analyses, although expensive, provide important insights into the molecular mechanisms driving tumor heterogeneity, metastatic potential, and therapy resistance. As such, they may be crucial for the optimal treatment selection and individual risk assessment [92].

### 3.6. Combining CTC Enumeration Methods with Other Approaches

The use of liquid biopsy facilitates the integration of CTC enrichment methods with the analysis of other circulating biomarkers. In the study conducted by Liang et al., CTCs were isolated with a CTCBIOPSY device based on 8 µm pore filtering, stained by Diff staining and counted [93]. Diff staining or Diff-Quick is often used for rapid onsite evaluation (ROSE) to estimate the percentage of malignant cells and their overall abundance [94]. Plasma was collected to extract cell-free DNA (cfDNA) from apoptotic or necrotic cancer cells for downstream evaluation of DNA methylation within selected genes. The study found that combining CTC detection with cfDNA methylation analysis improved the sensitivity and specificity of early diagnosis [93]. Such an integrated strategy may provide a wide range of information crucial for the efficient management of cancer patients.

## 4. Monitoring Anti-Lung Cancer Therapy

There are multiple treatment regimens available for lung cancer patients, depending on cancer status and patient condition. Nevertheless, their long-term safety and effectiveness remain limited. Moreover, due to cancer heterogeneity and clonal evolution, many tumors develop resistance mechanisms that compromise treatment outcomes. Therefore, numerous novel solutions are under development in clinical trials.

Various techniques for monitoring the progress of anticancer treatment have been developed over the years. They aim to guide clinicians in the management of cancer patients. A significant amount of the literature pinpoints CTC enumeration and characterization as a biomarker for disease prognosis and prediction of treatment response. The following sections will focus on the application of CTC count in monitoring different types of lung cancer therapies, including radiotherapy (RT), chemotherapy, tyrosine kinase inhibitors (TKIs), and immunotherapy.

### 4.1. Monitoring Radiotherapy

Radiotherapy (RT) in lung cancer is often used in combination with other methods as curative or palliative treatment. It is a frequent combinatory strategy with chemotherapy for inoperable NSCLC [95]. Conventional Fractional Radiotherapy (CFRT) has evolved to encompass several techniques, including anterior–posterior two-field RT, multi-field conformal RT, and intensity-modulated RT [96]. Throughout the years, radiotherapy has been refined to minimize side effects, primarily toxicity to surrounding healthy tissues. The development of four-dimensional computed tomography (4D CT) has improved the precision of tumor targeting. Furthermore, the stereotactic ablative body radiotherapy has been introduced to deliver higher ablative doses of radiation in fewer fractions, thereby improving efficacy and tolerability [97].

**Figure 3:**
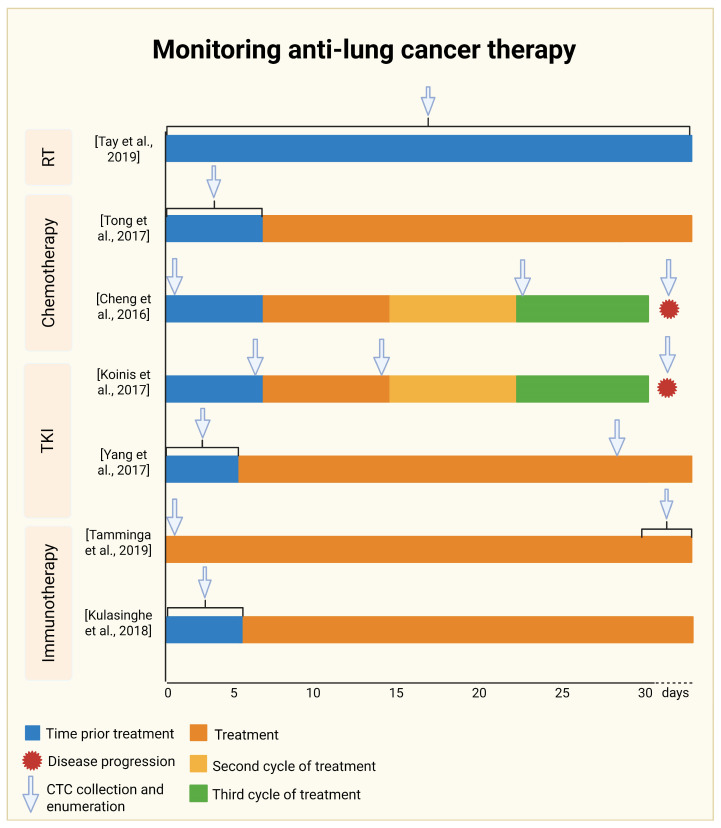
Lung cancer clinical trials utilizing CTC counts to predict treatment response. Schematic representation of treatment, CTC collection and enumeration of cited clinical trials on the timeline, divided into radiotherapy, chemotherapy, tyrosine kinase inhibitors (TKIs), and immunotherapy. The brackets above the bars represent period of time when samples were collected. Arrows show specific sampling point. Individual colors indicate time prior treatment and following cycles of anti-lung cancer therapy. Created in Biorender.com (http://BioRender.com/ckbrqki), accessed on 15 December 2025 [98,100,101,102,103,104,105].

The CONVERT study described by Tay and colleagues [98] provided key information on the prognostic value of CTCs and further monitoring the treatment of limited-stage SCLC (LS-SCLC) with chemoradiotherapy. Treatment consisted of Cisplatin, Etoposide, 45 Grays in 30 fractions twice a day or 66 Grays in 33 fractions daily [99]. Blood samples were collected before starting treatment as baseline samples to assess prognostic potential of CTCs (Figure 3). Then, CTCs were enriched using the CellSearch system from 7.5 mL blood samples. The threshold of 15 CTCs was chosen as an optimal cut-off for “favorable” or “unfavorable” LS-SCLC cases. The median overall survival (OS) was 26.7 months and 5.9 months for the “favorable” and “unfavorable” groups, respectively. The median progression-free survival (PFS) was 19 months for <15 CTCs detected, while for ≥15 CTCs it was 5.5 months. Two additional thresholds of CTC counts were also characterized: 2 and 50 CTCs. These thresholds also showed a correlation between CTC counts and OS and PFS. In summary, lower levels of CTCs were associated with higher values of PFS and OS, indicating that CTCs may serve as a predictive factor (Table 2 and Table S1) [98].

### 4.2. Monitoring Chemotherapy

Chemotherapy is one of the key treatment strategies, especially in advanced stages of lung cancer. Although this strategy has been employed for years (the first study on anticancer chemotherapy dates back to 1946) [106], it may have some limitations, including non-specific action, low bioavailability, the development of drug resistance, and numerous side effects. Commonly used chemotherapeutic agents for the treatment of NSCLC include such compounds as cisplatin, docetaxel, gemcitabine, and paclitaxel. In case of SCLC, chemotherapeutic regimens usually consist of doxorubicin or low-dose cisplatin [107]. However, novel chemotherapeutic regimens are still being developed, and CTC detection appears to be an attractive approach for determining their treatment potential (Table 2).

In the study by Tong et al. [100], the predictive significance of CTCs was analyzed in patients with NSCLC undergoing standard first-line platinum-based chemotherapy. The study included 127 patients with histologically confirmed advanced NSCLC (stage IIIb or IV). The Cyttel method was used for the CTC detection. In contrast to CellSearch, Cyttel is an EpcAM-independent technique based on immunomagnetic leukocyte depletion (using anti-CD45 antibodies). Further confirmation of CTC identity was established as follows: negative immunochemical staining for CD45, positive DAPI staining, and positive detection of chromosome 8 centrosome with FISH probes. In the lung cancer group, 73 of the 127 patients had their blood samples tested for the presence of CTCs before all three treatment phases. Peripheral blood (3.2 mL) was collected for analysis up to 7 days prior to chemotherapy administration (Figure 3). A cut-off threshold was established in relation to PFS and OS parameters at 8 CTCs per 3.2 mL of blood sample. No correlation was observed between clinicopathological parameters and high versus low CTC content. However, patients with higher CTC values before treatment had significantly shorter OS and PFS. In patients whose CTC count increased after the introduction of therapy (≥8 CTCs per 3.2 mL blood sample), the median OS was lower (12.0 months), compared to the patients whose CTC count decreased or did not change after the introduction of treatment (<8 CTCs per 3.2 mL of blood sample, median OS = 13.3 months, *p* = 0.009). Analogous results were obtained for PFS. For the group with ≥8 CTCs per 3.2 mL blood sample, the median PFS was 5.5 months, whereas the group with a lower CTC count—6.4 months [100].

In another study, Cheng and colleagues [101] investigated a group consisting of 91 patients with extensive-stage small-cell lung cancer (ES-SCLC) treated with etoposide-cisplatin or etoposide-lobaplatin. The CellSearch approach was applied for CTC enumeration at three time points: seven days prior to treatment, after the second cycle of chemotherapy, and at the time of disease progression (Figure 3). The cut-off value was established as 10 CTCs per 7.5 mL of blood sample based on time-dependent receiver operating characteristic (ROC) analysis. In the first stage of the trial (baseline), no statistically significant difference was found in the PFS parameter. However, all the following analyses were statistically significant. Median OS in the group of patients with high CTC count (≥10 CTCs per 7.5 mL of blood sample) was 8.2 months compared to the group of patients with the low CTC count (<10 CTCs per 7.5 mL of blood sample; median OS = 16.6 months). Following the second chemotherapy cycle, OS and PFS were more unfavorable for the cohort with high CTC content compared to the group of patients with low CTC levels (median PFS was 3.1 to 5.6 months; median OS was 6.9 to 12.7 months, respectively). In the third stage of the trial, the relapsed patients with a higher level of CTCs had a shorter median OS (8.3 months) compared to those with a lower level of CTCs (13.1 months). Furthermore, patients were assigned to subsequent cohorts based on the shifts in the number of CTCs between the first and second stages of the trial. Patients with a lower CTC count at both time points had a prolonged median PFS and median OS compared to patients with a higher CTC count at baseline and a reduced or high CTC number after the second cycle of chemotherapy. In the latter group (with high CTC number at both stages), median PFS and median OS were the shortest, indicating the worst prognosis. The above observations confirm that a change in CTC number in a blood sample may help assess the response to SCLC chemotherapy [101]. Analogous results were obtained from a clinical trial (NCT01638546) on a pooled cohort of 94 SCLC patients treated with either an alkylating agent, Temozolomide, and/or a poly(ADP-ribose) polymerase 1 (PARP) inhibitor (Veliparib). CTCs were enumerated with the CellSearch system in a 7.5 mL blood sample. The data showed that higher CTC counts at baseline and after the first treatment cycle were significantly associated with shorter median OS [108].

### 4.3. Tyrosine Kinase Inhibitor Therapy Monitoring

The most common target for tyrosine kinase inhibitor therapy in lung cancer is Epidermal Growth Factor Receptor (*EGFR*). *EGFR* gene mutations and overexpression occur frequently in NSCLC and remain one of the main factors contributing to the disease progression. There are several strategies that utilize EGFR-TKI. First-generation EGFR-TKIs, such as Gefitinib or Erlotinib, act as reversible inhibitors, while second-generation TKIs (Afatinib, Dacomitinib) bind irreversibly. The third generation of EGFR-TKIs, such as Osimertinib, is designed to target specific EGFRT790M mutation. This type of treatment is implemented for patients who are resistant to the first and second-generation TKIs. Fourth-generation TKIs are currently under development, aiming to overcome the acquired C797S mutation, which causes resistance to the third-generation TKIs [109]. Another druggable tyrosine kinase gene is anaplastic lymphoma kinase (*ALK*), as *ALK* rearrangements are detected in 3–7% patients with NSCLC [110]. Crizotinib is the first-generation ALK-TKI; however, new generation TKIs, such as Alectinib, Lorlatinib, or Brigatinib, provide superior efficacy [110]. Similar TKIs are also used in the treatment of lung cancer subtypes characterized by *ROS1* gene rearrangements, which are detected in approximately 1–2% NSCLC patients. First-line ROS1-TKIs include Crizotinib and Entrectinib. However, in resistant patients, Lorlatinib or Repotretynib may be employed as a second-line treatment [111].

The continued emergence of resistance mechanisms drives the development of novel therapies related to TKIs, and CTC enumeration appears to be one of the possible indicators of active disease (Table 2). Koinis and colleagues [102] analyzed the efficacy of Pazopanib therapy in 39 SCLC patients responsive to platinum-based treatment and 19 chemotherapy-resistant patients. Pazopanib is a multitarget TKI with additional anti-angiogenic activity specific against Vascular Endothelial Growth Factor Receptors 1–3 (VEGFRs 1–3), Platelet-derived Growth Factor Receptors α/ß (PDGFRs α/ß), and Proto-Oncogene C-KIT (c-Kit). CTC levels were measured as a potential marker of response to treatment. CTCs detected with the CellSearch system were enumerated before the first dose of Pazopanib, before the start of the second dosing cycle, and upon disease progression (Figure 3). The cut-off level was established as 5 CTCs per 7.5 mL of blood sample. The data indicated that administration of one cycle of Pazopanib significantly reduced the number of CTCs. At the beginning, 50% of cases showed ≥5 CTCs per 7.5 mL of blood sample. After one Pazopanib cycle, such values were detected only in 20% of cases. Moreover, patients with a partial response to treatment and stable disease showed a lower presence of CTCs compared to patients with disease progression. Additionally, a high CTC level was associated with shortened OS (5.2 months to 10.1 months) and PFS (1.9 months to 3.6 months). Median OS was also higher in patients with no detectable CTCs after the first cycle, compared to patients with present CTCs. Results were statistically significant, confirming that CTCs may be utilized as a predictive marker in lung cancer treatment with TKIs [102].

In another study, 107 NSCLC patients with an EGFR mutation in stage IIIB or IV disease were treated with Erlotinib or Gefitinib [103]. CTCs were counted by CellSearch at two time-points: within 5 days before treatment and on day 28 of treatment (Figure 3). The CTC number cut-off was established as 5 CTCs per 7.5 mL of blood sample. Results showed that, at baseline, 44% of patients had ≥2 CTCs detected in their blood sample, with counts ranging from 2 to 80. Among them, 29% of patients had 2–4 CTCs (defined as the favorable group), while 15% of patients had ≥5 CTCs per blood sample (unfavorable group). Median PFS was significantly improved in the group with a baseline of 0–4 CTCs per 7.5 mL of blood sample (11.1 months) compared to the group with a higher CTC count (6.8 months). The median PFS for patients with 0–4 CTC counts on day 28 of treatment was significantly longer (11.6 months) than for patients with higher levels of CTCs (6.3 months). Furthermore, a drop in CTC count during treatment was associated with prolonged PFS. According to multivariate analyses, the value of ≥5 CTCs per blood sample on day 28 of treatment was the strongest prognostic indicator of poor PFS, with a hazard ratio (HR) of 8.017 [103]. The same researchers conducted a clinical trial on NSCLC patients with the EGFRT790M mutation treated with AZD9291 (the third-generation TKI). A similar CTC counting regimen was applied as a marker for therapy response. The data indicated prolonged PFS in patients with a favorable CTC count of <5 CTCs compared to ≥5 CTCs. This outcome was significant both at baseline (9.3 versus 6.5 months as assessed in 68 patients) and on day 28 of treatment (9.7 versus 6.2 months as shown in 55 patients). The multivariate analysis demonstrated that higher CTC count at the beginning of therapy remains the most significant prognostic marker associated with high risk of progression (HR = 6.835) [112].

### 4.4. Monitoring Immunotherapy

Immunotherapy is a rapidly developing field in cancer treatment. A huge breakthrough was the development of therapies based on immune checkpoint inhibitors (ICIs). Another immunotherapeutic strategy includes cancer vaccines, monoclonal antibodies, and CAR (Chimeric Antigen Receptor)-T therapy [113]. In the context of lung cancer, the main ICIs include PD-1/PD-L1 (Programmed cell death protein 1/Programmed death-ligand 1) and CTLA-4 (Cytotoxic T-lymphocyte-associated protein 4) inhibitors. The inhibitors of the PD-1/PD-L1 axis, such as Nivolumab, Pembrolizumab, or Atezolizumab, impair the interaction between PD-1 and its ligand, thereby restoring T cell activity and preventing exhaustion. CTLA-4 inhibitors, such as ipilimumab or Tremelimumab, enhance the activation and function of T lymphocytes [3]. Tumor-specific vaccines also show therapeutic potential; however, these solutions are still in the early stages of development. These include peptide/protein vaccines (e.g., CIMAvax-EGF) [114], vector vaccines (TG4010) [115], or allogeneic vaccines (Belagenpumatucel-L) [116]. The major advantage of immunotherapy lies in its purposeful action, durable therapeutic response, effectiveness in a vast patient population, and fewer side effects. However, the efficacy of ICI treatment may be impaired due to resistance and immunosuppression. Thus, much effort is implemented to develop novel, more effective treatment regimens and monitoring tools [3].

Tamminga and colleagues [104] conducted a study on 104 advanced NSCLC patients (IIIB—11% and IV stage—89%) treated with various ICI regimens (89% Nivolumab as monotherapy, 8% Pembrolizumab, 5% Atezolizumab, 2% Ipilimumab and Nivolumab). Blood samples, analyzed with the CellSearch system, were collected at two time points: at the beginning of treatment (T0) and after 4–6 weeks of treatment (T1) (Figure 3). The presence of CTCs, as detected in 33 of 104 patients (32%), showed a significant prognostic potential. Median PFS in cases with CTCs ≥ 1 was established as 1.4 months, compared to cases without CTCs (median PFS = 4.8 months), whereas median OS was 4.5 months compared to 12.1 months, respectively. At 4–6 weeks of treatment (T1), samples were successfully collected from 63 of 104 patients. The remaining 41 patients were excluded from the study due to disease progression, death, issues with sample collection, or withdrawal from the study. CTCs were detected in 17 of 63 patients (27%). Results showed that out of these 17 patients, 8 (47%) had no CTCs at T0, prior to the initiation of treatment. Interestingly, six patients who were positive for CTCs at T0 had no CTCs after treatment. CTC level decreased in 11 cases after treatment, increased in another 11 cases, whereas no change in CTC number was observed in 41 patients. Elevated CTC number after treatment was significantly associated with shorter PFS (HR = 4.46; *p* < 0.01) and OS (HR = 2.4; *p* = 0.04). Overall, these observations suggest that the presence of detectable CTCs before and after treatment may serve as an indicator of poor prognosis [104].

To select patients who may be more responsive to anti-PD-L1 therapy, Kulasinghe and colleagues [105] utilized CTCs to predict disease progression in patients with stage IV NSCLC prior to Nivolumab administration (Figure 3). The study qualification criteria envisaged positive PD-L1 expression—a marker associated with a better response to immunotherapy. The ClearCell FX system, equipped with a CTChip, was used to quantify CTCs based on size and deformability differences between CTCs and hematopoietic cells. CTCs were detected in 17/33 (51.5%) NSCLC patients, with a range of 1 to 28 CTCs and/or a CTC cluster per 3.75 mL of blood sample. PD-L1 expression on CTCs was identified in 11 of 17 cases (64.7%). Patients with detectable CTCs demonstrated a non-insignificant trend towards an increased risk of disease progression (HR = 2.246, *p* = 0.06). The authors noted that the small sample size and advanced disease stage were limiting factors, emphasizing the need for further research [105]. Another study on pembrolizumab treatment also revealed the predictive potential of CTC in the NSCLC cohort. The data demonstrated a significantly higher CTC count (as detected with the CellSearch system) in patients with disease progression compared to those with complete or partial response [117].

### 4.5. Meta-Analysis

The comprehensive meta-analysis published by Jin and colleagues [11] provided essential conclusions for the prognostic value of CTC detection in the context of lung cancer. The articles cited in this meta-analysis included 27 studies involving a total of 2957 patients with lung cancer who received chemotherapy, immunotherapy, or radiotherapy. Clinical trials were selected based on the following criteria: CTC level was assessed as an outcome predictor, OS and/or PFS were determined. The authors excluded replicated reports based on the same cohort, trials without sufficient data to estimate HR and 95% confidence intervals, or with an insufficient number of patients (<20). Pooled analysis delivered promising conclusions. A strong negative prognostic effect of the presence of CTCs was confirmed (regardless of the enumeration method and cut-off applied). In other words, an increased number of CTCs correlated with a worse prognosis.

Further in-depth statistical analyses revealed that CTC detection in SCLC has a stronger prognostic effect compared to NSCLC (HR 3.11 versus 2.11, respectively). These results reflect the higher metastatic potential of SCLC. Moreover, prognostic values varied depending on the selected markers. Detection of epithelial CTCs based on the presence of EpCAM or cytokeratin expression indicated a worse prognosis compared to epithelial–mesenchymal CTC hybrids that were also positive for vimentin or N-cadherin. Furthermore, prognostic potential was also influenced by the cut-off point established in trials. The prognostic effect was higher when the cut-off was established as ≥2 CTCs per 7.5 mL of blood sample (50.7% detection rate) compared to 1 CTC (36.3% detection rate). Thus, the authors concluded that higher detection thresholds correlate with a more accurate prognostic value of CTCs. Moreover, detection of CTCs in early stages of NSCLC may indicate vascular invasion and potential micrometastases. Detection of CTCs in I-III stage NSCLC was more related to worse OS (HR = 2.79) compared to III-IV stage (HR = 2.04). However, the authors pinpointed certain shortcomings of their meta-analysis that should be considered. These limitations include moderate heterogeneity across the described studies, attributed to the use of different markers and methods for CTC identification, cut-off values, and histological subtypes of lung cancer. Nevertheless, the authors concluded that CTC detection constitutes a universal indicator of unfavorable prognosis in lung cancer, regardless of the method used and CTC phenotype [11]. Similar associations between increased posttreatment CTC levels and poor patient outcomes were also demonstrated in other meta-analyses performed on lung cancer cohorts [10,118].

## 5. Limitations of Technologies for CTC Enumeration

Although the studies described above highlight a correlation between increased CTC count and shortened OS and PFS, the use of CTCs to monitor anticancer therapy still has its drawbacks. First of all, CTCs exhibit a high level of deformability, heterogeneity, and a dynamic EMT phenotype. These features may impair the detection sensitivity of available enrichment methods, as well as the technical feasibility and accuracy of CTC identification [28]. Another critical obstacle to the widespread use of CTC enumeration technologies is the low level of CTCs in the bloodstream, especially at the early stage of disease, which requires a larger volume of blood sample [36,64]. Both impediments lead to a key issue—a lack of a unified critical threshold for CTC counts that clearly distinguishes between favorable and unfavorable outcomes. In many studies, the values differ considerably, which may render the interpretation of some results questionable. Finally, the most informative data could be obtained through a comprehensive molecular characterization of individual CTCs, such as utilizing single-cell sequencing. However, this is an expensive approach, and as such, has limited applicability in the clinical setting [119].

## 6. Conclusions and Future Perspectives

In summary, CTCs provide a range of information related to tumor biology, including phenotype, biological features, genetic status, and the expression of tumor-specific markers. Growing evidence suggests that the presence of CTCs is significantly associated with poor prognosis, thereby meeting the criteria of a suitable marker for monitoring anticancer therapy in lung cancer. The use of liquid biopsy-derived CTCs from a patient has many advantages. Firstly, it enables early detection, real-time monitoring, and assessment of the temporal heterogeneity of cancer. Furthermore, CTCs have certain superior features compared to ctDNA or exosomes in monitoring therapy in lung cancer. CTCs, as whole cells, compared to ctDNA, carry more information, and may reflect dynamic biological changes, including resistance mechanisms or tumor heterogeneity. Moreover, CTCs may be utilized as a significant prognostic and predictive marker, regardless of the treatment type or molecular subtype of cancer. Apart from the enumeration procedure, current clinical trends appear to utilize CTCs for the profiling of molecular signatures and vulnerabilities of residual tumor cells to guide personalized patient care [120,121,122]. Nonetheless, CTCs also have certain limitations, including insufficient specificity, sensitivity, and standardization of enumeration protocols, as well as relatively high operational costs. Therefore, these elements require improvement to introduce liquid biopsy-derived CTCs into widespread and routine use in therapy monitoring. To exploit CTC potential as a prognostic and predictive marker in lung cancer, it is crucial to refine and standardize operating procedures and recommendations for CTC collection timing, methodological approaches, and cut-off points across different subtypes of lung cancer and treatment strategies. Large-scale, multicenter collaborative efforts with harmonized protocols, merged data, and cross-validation would provide significant benefits for the future application of CTCs within clinical context. Notably, it is crucial to address the technical challenges of CTC enrichment methods, which have the potential to revolutionize the evaluation of treatment response in lung cancer.

## Figures and Tables

**Table 1:** Comparison of different CTC enrichment methods (CellSearch, Dynabeads, SPPCNs, Dielectrophoresis, Parsortix) based on detection marker(s)/feature(s).

Enrichment Method	Detection Marker(s)/Feature(s)	Advantages	Limitations	Specificity *	Sensitivity (% of Patients with Detected CTC/CTCs) *
CellSearch	EpCAM, DAPI, CK8, CK18 and/or CK19 (+) CD45 (−)	Standardization, reproducibility, analytical performance, flexibility in preparation, wide range of clinical applications	Hard to detect CTCs in early stages of disease, unable to detect CTCs with lost EpCAM expression during EMT	99.7% (≥2 CTCs/7.5 mL of blood sample) [68]	20% metastatic lung cancer patients [68]
Dynabeads	Magnetic potential + anti-EpCAM antibody + RT-PCR assay for CK20 expression	Easy and fast isolation, high purity and efficiency, precise particle binding, easy automatization	Unable to detect CTCs with lost expression of EpCAM during EMT	100% [69]	10^3^ spiked-in HT29 cells (200 cells/mL) in 5 mL; 28% colorectal cancer patients [69]
Superparamagnetic Positively Charged Nanoparticles (SPPCNs)	Negative surface charge of CTCs (magnetic potential) + immunofluorescence or iFISH (DAPI, EpCAM or CD45)	EpCAM-independent in the first stage	Cellular structure disturbances, need for buffer optimization	100% [70]	2–8 CTCs/1 mL of blood sample; 100% colorectal cancer patients [70]
Dielectrophoresis	Physical features: size, nuclear morphology, cell membrane morphology, dielectric properties of cells, and their membrane surface	Non-invasive, epitope independence	Requires precise control of physical conditions	not analyzed [71]	66–84% (including epithelial-, EMT-, and CSC-CTCs) depending on the treatment status in breast cancer patients [71]
Parsortix	Cell size and compressibility	Low cost, simple operation, epitope independence	Similar size of CTCs and leukocytes	93.1% [72]	1 CTC/average 8.6 mL; 45.3% metastatic breast cancer patients [72]
Atomic force microscopy (AFM)-compatible microfluidic platform	EpCAM, PSA, PSMA, DAPI, CK (+) + AFM	External access to intact cells, determination of the elasticity, deformation, and cell adhesion forces	High cost, slow process, EpCAM dependent	AFM analysis of EpCAM-captured CTCs showed decreased stiffness and adhesin expression in case of metastatic prostate cancer compared to localized cancer. No specificity and sensitivity was evaluated for AFM method [73].	

* Specificity and sensitivity may vary due to different studies and protocols.

**Table 2:** Characterization of clinical trials applying CTC count as a prognostic and predictive marker. The table provides information on the disease type and stage, form of treatment, method of CTC enrichment, cut-off thresholds, and results correlating CTC count with clinical outcomes (OS, PFS). The following equipment was used in these trials: CellSearch [Veridex LLC (Raritan, NJ, USA)], Cyttel [Cyttel Bioscences Co., Ltd. (Beijing, China)], ClearCell FX with CTChip (Clearbridge, Singapore). The arrow in the Result column denote higher (↑) and lower (↓) level of the mentioned parameters. The abbreviation n.a. (not applicable) is included if the NCT clinical trial number is not available for a cited study.

NCT/Reference	Disease (Stage)	Drug (Treatment)	CTC Enrichment Method	Cut-Off Threshold (CTCs/mL of Blood Sample)	Results
NCT00433563/[98]	Limited stage (LS)-SCLC	Cisplatin, Etoposide, 45 Grays in 30 fractions twice a day or 66 Grays in 33 fractions daily (Chemoradiotherapy)	CellSearch	15 CTCs/7.5 mL blood samples	↑ amount of CTC = ↓ time of OS and PFS
n.a./[100]	NSCLC (IIIB/IV)	Platinum (Chemotherapy)	Cyttel + FISH/immunofluorescence	8 CTCs/3.2 mL blood samples	↑ amount of CTC = ↓ time of OS and PFS
n.a./[101]	Extensive stage (ES)-SCLC	Etoposide-Cisplatin/Etoposide-Lobaplatin (Chemotherapy)	CellSearch	10 CTCs/7.5 mL blood samples	↑ amount of CTC = ↓ time of OS and PFS
NCT01253369/[102]	Chemotherapy-resistant SCLC	Pazopanib (Tyrosine kinase inhibitor therapy)	CellSearch	5 CTCs/7.5 mL blood samples	↑ amount of CTC = ↓ time of OS and PFS
n.a./[103]	NSCLC with EGFR mutation (IIIB/IV)	Erlotinib/Gefitinib (Tyrosine kinase inhibitor therapy)	CellSearch	5 CTCs/7.5 mL blood samples	HR = 8.017 for ↓ PFS in day 28 of treatment in samples with ≥5 CTCs
n.a./[104]	NSCLC (IIIB/IV)	Nivolumab/Pembrolizumab/Atezolizumab/Ipilimumab and Nivolumab (Immunotherapy)	CellSearch	Detection of single CTC/7.5 mL blood samples	↑ amount of CTCs and ↓ time of OS (HR = 2.4) ↑ amount of CTCs and ↓ time of PFS (HR = 4.46)
n.a./[105]	NSCLC (IV)	Nivolumab (Immunotherapy)	ClearCell FX with CTChip	Detection of single CTC/3.75 mL blood samples	*p*-value indicated that the results were not statistically significant

## Data Availability

No new data were created or analyzed in this study. Date sharing is not applicable to this article.

## Supplementary Materials

The following supporting information can be downloaded at: https://www.mdpi.com/article/10.3390/ijms27010384/s1.

## Abbreviations

The following abbreviations are used in this review:

AFM	Atomic force microscopy
AFM-FS	AFM-force spectroscopy
AFM-IR	Infrared nanospectroscopy
ALK	Anaplastic lymphoma kinase
CAFs	Cancer-associated fibroblasts
CAR	Chimeric Antigen Receptor
cfDNA	Cell-free DNA
CFRT	Conventional Fraction Radiotherapy
CK18	Caspase-cleaved cytokeratin 18
c-Kit	Proto-Oncogene C-KIT
CTCs	Circulating tumor cells
ctDNA	Circulating tumor DNA
CTLA-4	Cytotoxic T-lymphocyte-associated protein 4
CXCL12	C-X-C Motif Chemokine Ligand 12
DALYs	Disability-Adjusted Life Years
DEP	Dielectrophoresis
DLD	Deterministic lateral displacement
EGF	Epidermal growth factor
EGFR	Epidermal growth factor receptor
E/M	Epithelial and mesenchymal states
EMT	Epithelial–mesenchymal transition
EpCAM	Epithelial Cell Adhesion Molecule
ES-SCLC	Extensive-stage small-cell lung cancer
FDA	Food and Drug Administration
FISH	Fluorescence in situ hybridization
GBD	Global Burden of Disease
HR	Hazard ratio
HS-AFM	High-Speed AFM
ICAM-1	Intercellular adhesion molecule 1
ICIs	Immune checkpoint inhibitors
IF1	Inertial focusing stage 1
IF2	Inertial focusing stage 2
iFISH	Immunostaining fluorescence in situ hybridization
LS-SCLC	Limited-stage SCLC
MACS1	Magnetically activated cell sorting stage 1
MACS2	Magnetically activated cell sorting stage 2
M-CSF	Macrophage-Colony Stimulating Factor
MDSCs	Myeloid-derived suppressor cells
MET	Mesenchymal–epithelial transition
n.a.	Not applicable
nDEP	Negative dielectrophoresis
NETs	Neutrophil extracellular traps
NFκB	Nuclear Factor Kappa B
NGS	Next-Generation Sequencing
NK	Natural Killer
NSCLC	Non-small cell lung cancer
OS	Overall survival
OXPHOS	Oxidative phosphorylation
qPCR	Quantitative Polymerase Chain Reaction
PARP	Poly(ADP-ribose) polymerase 1
PCR	Polymerase chain reaction
PD-1	Programmed Cell Death Protein 1
pDEP	Positive dielectrophoresis
PD-L1	Programmed Cell Death-Ligand 1
PDGFRs α/ß	Platelet-derived Growth Factor Receptors α/ß
PDMS	Poludimethylsiloxane
PEI	Poly(ethyleneimine)
PFS	Progression-free survival
PI	Poly(ethyleneimine)
PMN-MDSCs	Polymorphonuclear-myeloid derived suppressor cells
PSA	Prostate-Specific Antigen
PSMA	Prostate-Specific Membrane Antigen
ROC	Receiver Operating Characteristic
ROS	Reactive oxygen species
ROSE	Rapid onsite evaluation
RT	Radiotherapy
SCLC	Small cell lung cancer
SCS	Single Cell Sequencing
Snail1/2	Snail Family Transcriptional Repressors 1/2
SPPCNs	Superparamagnetic positively charged nanoparticles
TBL	Trachea, bronchus, and lung
TDLNs	Tumor-Draining lymph nodes
TGF-β	Transforming growth factor-β
TKIs	Tyrosine kinase inhibitors
TME	Tumor microenvironment
TWIST	TWIST family of basic helix–loop–helix factors
VEGFR 1–3	Vascular Endothelial Growth Factor Receptor 1–3
YLDs	Years Lived with Disability
YLLs	Years of Life Lost
ZEB1/2	Zinc-finger E-box-binding homeobox factors 1/2
